# Supplementary material for: A systematic review and realist synthesis on toilet paper hoarding: COVID or not COVID, that is the question
Source: PeerJ. 2021 Jan 29;9:e10771. doi: 10.7717/peerj.10771 (PMC7849510; doi:10.7717/peerj.10771)
Supplement: Supplemental Information 2 [file peerj-09-10771-s002.doc]

**Table S1. Risk of bias assessment for studies included in the Systematic Review (n=6) (Murad et al., 2018)**

| **Tools for evaluating methodological quality of case reports** | | **Case reports** | | | | | |
| --- | --- | --- | --- | --- | --- | --- | --- |
| **Domains** | **Leading explanatory questions** | **Case 1**  Klimke et al., 2016 | **Case 2**  Sauvageau and Yesovitch, 2006 | **Case 3**  Saint-Martin et al., 2007 | **Case 4**  Saint-Martin et al., 2012 | **Case 5**  Fisher et al., 2014 | **Case 6**  Chisholm and Martin, 1981 |
| Selection | 1. Does the patient(s) represent(s) the whole experience of the investigator (centre) or is the selection method unclear to the extent that other patients with similar presentation may not have been reported? | Yes | Yes | Yes | Yes | Yes | Yes |
| Ascertainment | 2. Was the exposure adequately ascertained? | Yes | Yes | Yes | Yes | Yes | Yes |
|  | 3. Was the outcome adequately ascertained? | Yes | Yes | Yes | Yes | Yes | Yes |
| Causality | 4*. Were other alternative causes that may explain the observation ruled out? | No | No | No | No | No | No |
|  | 5*. Was there a challenge/rechallenge phenomenon? | No | No | No | No | No | Yes |
|  | 6*. Was there a dose-response effect? | No | No | No | No | No | No |
|  | 7. Was follow-up long enough for outcomes to occur? | Yes | No | Yes | No | No | Yes |
| Reporting | 8. Is the case (s) described with sufficient details to allow other investigators to replicate the research or to allow practitioners make inferences related to their own practice? | No | Yes | Yes | Yes | Yes | Yes |
| **Total scores** | Max. 8 | 6 | 6 | 6 | 6 | 6 | 7 |

*Questions 4, 5 and 6 are particularly relevant for cases reporting adverse drug events. Total scores are an overall judgement about methodological quality and not the total sum of the 8 items.
