# Supplementary figures and images for: A systematic review and realist synthesis on toilet paper hoarding: COVID or not COVID, that is the question
Source: PeerJ. 2021 Jan 29;9:e10771. doi: 10.7717/peerj.10771 (PMC7849510; doi:10.7717/peerj.10771)

**Figure S1. Google trend topics for the terms ‘toilet paper’ and ‘covid’ during the first 7 months of 2020.**

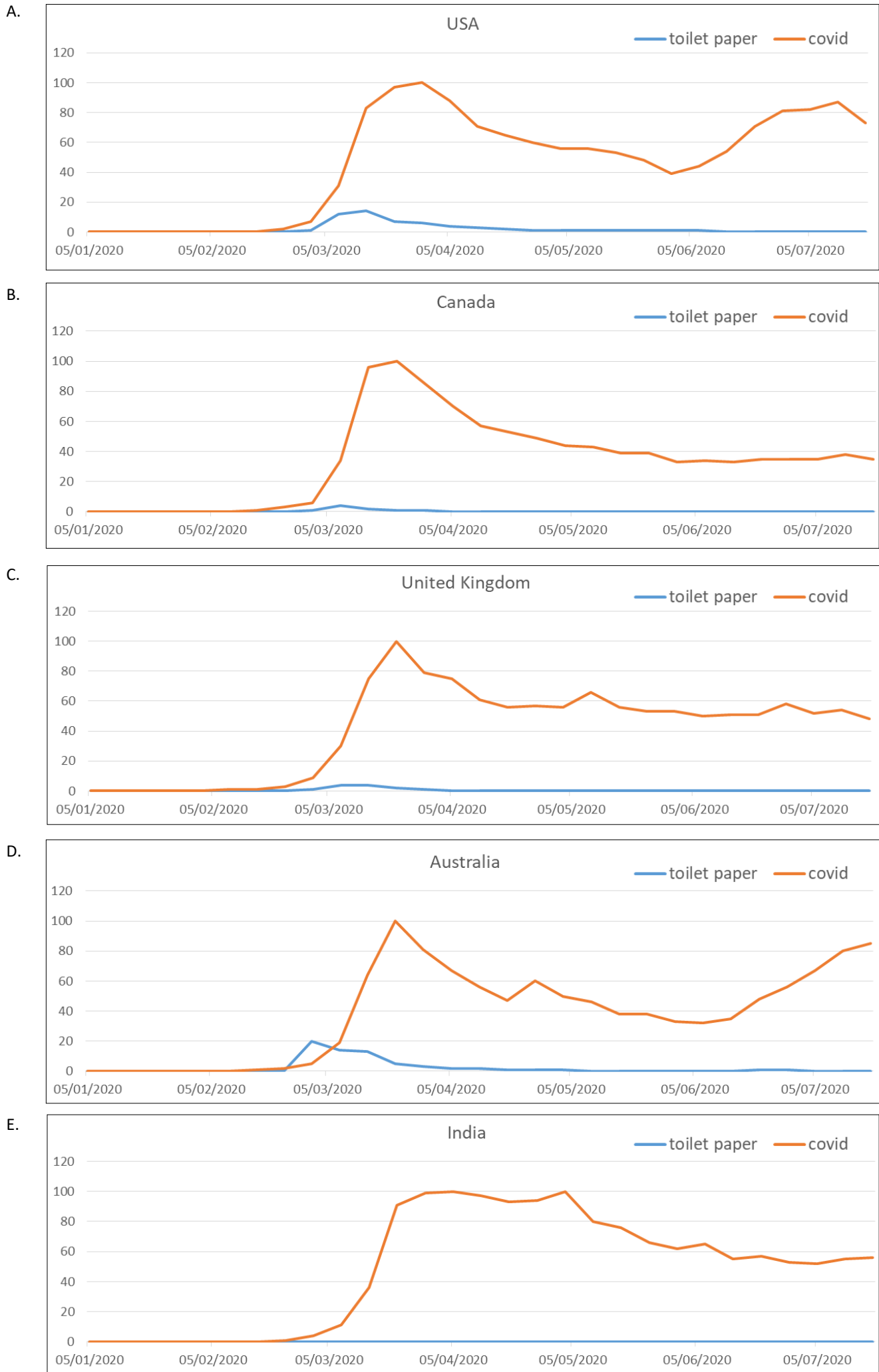

Supplement: Supplemental Information 3 [file peerj-09-10771-s003.pdf]
