# Supplementary material for: A systematic review and realist synthesis on toilet paper hoarding: COVID or not COVID, that is the question
Source: PeerJ. 2021 Jan 29;9:e10771. doi: 10.7717/peerj.10771 (PMC7849510; doi:10.7717/peerj.10771)
Supplement: Supplemental Information 4 [file peerj-09-10771-s004.docx]

**The rationale for conducting the systematic review / meta-analysis**

In the last months, the coronavirus disease 2019 (COVID-19) pandemic has had negative effects on mental health and stress-related behaviours. A particular conduct was toilet paper hoarding in the weeks following worldwide lockdowns. As this seemed to be an unprecedented behaviour, we aimed to study the evidence on the COVID-19 pandemic and toilet paper hoarding with a particular aim to identify which risk factors (including COVID-19-related factors, stress-related situations, personality traits, or mental health problems) are associated with the risk of toilet paper hoarding. We conducted a systematic review and realist synthesis of the recent evidence on this topic.

**The contribution that it makes to knowledge in light of previously published related reports, including other meta-analyses and systematic reviews**

Our study confirms that the COVID-19 pandemic has been associated with a worldwide increase in toilet paper hoarding. Social media and social cognitive biases seem be major contributors to this hoarding behaviour and might explain some differences in toilet paper hoarding between countries. Other mental health-related factors, such as the stressful situation of the COVID-19 pandemic and fear of contagion, or particular personality traits (conscientiousness) are likely to be involved.
